# Post translational modifications of milk proteins in geographically diverse goat breeds

**DOI:** 10.1038/s41598-021-85094-9

**Published:** 2021-03-10

**Authors:** P. K. Rout, M. Verma

**Affiliations:** Genetics and Breeding Division, ICAR-Central Institute for Research on Goats, Makhdoom, Farah, Mathura, Uttar Pradesh 281122 India

**Keywords:** Biochemistry, Biotechnology, Molecular biology

## Abstract

Goat milk is a source of nutrition in difficult areas and has lesser allerginicity than cow milk. It is leading in the area for nutraceutical formulation and drug development using goat mammary gland as a bioreactor. Post translational modifications of a protein regulate protein function, biological activity, stabilization and interactions. The protein variants of goat milk from 10 breeds were studied for the post translational modifications by combining highly sensitive 2DE and Q-Exactive LC-MS/MS. Here we observed high levels of post translational modifications in 201 peptides of 120 goat milk proteins. The phosphosites observed for *CSN2, CSN1S1, CSN1S2, CSN3* were 11P, 13P, 17P and 6P, respectively in 105 casein phosphopeptides. Whey proteins *BLG* and *LALBA* showed 19 and 4 phosphosites respectively. Post translational modification was observed in 45 low abundant non-casein milk proteins mainly associated with signal transduction, immune system, developmental biology and metabolism pathways. P_asp_ is reported for the first time in 47 sites. The rare conserved peptide sequence of (SSSEE) was observed in αS1 and αS2 casein. The functional roles of identified phosphopeptides included anti-microbial, DPP-IV inhibitory, anti-inflammatory and ACE inhibitory. This is first report from tropics, investigating post translational modifications in casein and non-casein goat milk proteins and studies their interactions.

## Introduction

Milk is the primary source of nutrition for mammals and serves as a major vehicle of maternal immunity transfer, thus, plays a vital role in inclusive development of the neonates. The milk proteome is extremely complex due to abundant post-translational modifications and various proteolytic processes^1^. Milk protein composition exhibited high heterogeneity due to numerous genetic variants, and isoforms with different degrees of posttranslational modifications such as phosphorylation and glycosylation in caseins^2,3^. Milk proteins exhibit conformational structure due to post translational modifications and constitutive levels of proteolytic activity produce a range of significant peptides. The posttranslational modifications of the polypeptide chain occur in the Golgi apparatus of the mammary epithelial cells^4^. Casein phosphorylation at amino acid serine or threonine is catalyzed by kinase enzymes^5^. Phosphorylation is affected by different factors such as protein sequence, efficacy of kinase enzymes, gene expression, substrate availability and access to phosphorylation site which is responsible for the specific protein conformation^2,6^.

Protein functions such as binding, stabilization, biological activity, interactions with proteins and other biomolecules are regulated by phosphorylation-dephosphorylation of protein^7^. Phosphorylation stabilizes calcium phosphate nano clusters in casein micelles^8^. The micellar structure of casein enables milk to carry calcium and phosphate to the neonate by channelizing the risk of mammary gland bio-calcification^9,10^. Phosphorylation state of caseins varies widely from 1P to 3P on *CSN3*, 4P to 5P on *CSN2*, 8P to 9P on *CSN1S1*, 10P to 13P on *CSN1S2*^6,11^. In bovine milk, *CSN1S1* accounts for about 35% of the total casein and has 2 common phosphorylation isoforms: *CSN1S1*-8P and *CSN1S1*-9P. Similarly, *CSN1S2* accounts for about 10% of the total casein and is present with isoforms from 10 to 14P and occasionally with 9P or 15P^2,12^.

The identification and analysis of phosphopeptides has been challenging because of the relatively low stoichiometry, inherent lower ionization efficiency and variation of phosphorylation sites^13,14^. However, advances in proteomics have largely enhanced the quotient of protein identification. Phosphorylation of milk proteins has been studied in llama^15^, camel^16^ and goat milk fat globule membrane proteins^17^ using MS/MS proteomic approach. A total of 8 phosphopeptides corresponding to 18 phosphorylation sites were identified in *CSN1S1*, *CSN1S2* and *CSN2* of goat milk using nLC-MS/MS^18^. Casein phosphopeptides in goat milk have been studied by Olumee-shabon and Boehemer^18^ and phosphoproteome of goat milk fat globule membrane have been reported by Henry and others^17^. Phosphorylation have been reported for bovine caseins using inductively coupled plasma mass spectrometry (ICP-MS)^19^, equine *CSN1S1* and and *CSN2* by nESI-MS/MS^20,21^, donkey *CSN2* by MALDI-TOF and nESI-MS/MS^22^ and for *CSN1S1* and *CSN2* of water buffalo by MS^23^.

Bovine milk is extensively used, due to its high biological value and plasticity as it can be transformed to cheese and several other dairy products. The functional knowledge of casein and whey proteins has been identified for the presence of bioactive peptides^24^. These peptides show various biological activities and are released by proteolytic digestion of caseins and milk proteins in gut or during fermentation. This study was designed to gain an insight into molecular diversity of goat milk proteins and to identify various degree of PTM in casein and non-casein proteins. The characterization of high and low abundant milk proteins by gel based proteomic approach i.e. 2DE and nLC-MS/MS. The present study analysed post-translational modification such as phosphorylation, oxidation, and carbamidomethylation on casein and non-casein proteins in goat milk. As post-translational modifications (PTMs) are acting as the major means of intracellular communication, therefore, the interactions of various proteins involved in post translational modifications were analysed.

## Results

The present study has analyzed the post translational modifications in low and high abundant proteins in goat milk of genetically diverse goat breeds/genotypes reared in varied ecological and grazing condition in India. The present study generated a comprehensive profile of PTM in goat milk and identified PTM sites in relation to sample variations.

### The goat milk proteome

The milk samples from 10 Indian goat breeds were investigated regarding their genetic variation, proteome composition and post translational modifications. The selected populations were mapped in their home tract and reared in semi-intensive system and the animals were apparently healthy. Altogether 1240 milk samples were analyzed by means of 14% SDS-urea-PAGE and different allelic combinations of caseins were resolved to identify the protein variants. Further, the selected variants were analyzed by combining highly sensitive 2DE and Q-Exactive nLC-MS/MS using Thermo Fisher in-house reference database. Distinctive protein spots (n=144) selected from 21 variants in 2DE gels were processed by nLC-MS/MS. The MS data have been deposited to the ProteomeXchange Consortium via the PRIDE partner repository with the dataset identifier PXD013593. The .raw files generated from nLC-MS/MS of goat milk proteins were analysed for Sequest search using Proteome Discoverer (v2.2). By comparing the databases, a milk proteome of 578 peptides for 348 genes (800 Uniprot accessions) were identified using reference database. The post translational modifications were identified in 201 peptide sequences for 86 proteins (120 Uniprot KB accessions).

### Post translational modifications in goat milk proteins

The annotations of peptides observing modifications including cysteine carbamidomethylation, oxidation, acetylation and phosphorylation in each protein are presented in Table S2. Post translational modifications were observed in a total of 201 peptide sequences corresponding to 120 Uniprot ID across the reference databases. PTMs were observed in high and low abundant milk proteins in each breed. High performance Q-Exactive LC-MS/MS analyses identified 287 sites of phosphorylation on 120 unique phosphoproteins. The Ser/Thr/Tyr/Asp ratio was 128:70:42:47, respectively. The phosphorylation at Asp residues is reported for the first time in goat milk proteins.

#### Phosphorylation of high abundance proteins

The observed phosphosites for the high abundance proteins in goat milk are presented in Table 1. In the casein fraction, β-casein (*CSN2*) showed a maximum of 17 phosphosites, 4D, 5T, 6S and 2Y ; α-S2 casein (*CSN1S2)* showed 13 phosphosites (7S, 1D, 5T) ; α-S1 casein (*CSN1S1*) showed 11 phosphosites (5S, 3D, 1T, 2Y) ; and κ-casein (*CSN3)* showed 6 phosphosites (3S, 1Y, 2T), respectively. Whey proteins β-lactoglobulin (*BLG)* (P02756) showed 19 phosphosites (7D, 3Y, 5T, 4S) and α-lactalbumin (*LALBA*) showed 4 phosphosites (3D, 1Y).

**Table 1:** Identified phosphosites for the major milk proteins in *Capra hircus* reference database.

Protein name	Gene ID	Number of phosphosites identified	Identified phosphorylation sites
α-S1 casein	CSN1S1	11	S27, D58, S61, S63, T64, D66, Y95, D100, Y106, S130, S138
α-S2-casein	CSN1S2	13	S23, S24, S25, S145, T146, S147, S151, T154, D156, S159, T160, T161, T164
β-casein	CSN2	17	S50, T56, D58, D62, Y129, T135, S137, S139, T141, T143, D144, S157, S167, T169, S181, D197, Y206
κ-casein (Fragment)	CSN3	6	S24, Y16, S60, T64, T73, S95
α-lactalbumin	LALBA	4	D82, D83, D116, Y122
β-lactoglobulin	BLG	19	D29, T36, Y38, S39, S45, D46, S48, D51, S54, Y60, T67, T94, D114, T115, D116, Y117, T143, D147, D155

The phosphorylated peptides generated from these proteins showed different isoforms and are presented in Table 2. The phosphorylated peptide 76–93 in *CSN1S1* showed 5P at S_78_-S_82_ and peptide 58–73 showed 1P or 3P resulting in isoforms 10P or 12P. Similarly, peptides from *CSN1S2* showed variations leading to different isoforms. Peptides 19–40, 19–37 showed 3 variations in site of phosphorylation such as 1P S_24_ or 2P S_24,_ S_25_ or 3P S_23_, S_24_, S_25_. Peptide 142–153 showed 1P S_145_ or 1P S_147_ or 3P S_145_, T_146_, S_147_ and peptides 154–165/166 showed 1P S_159_ or 2P S_159_, T_160_ resulting in 8P isoform. β-casein peptides showed variations 1P or 6P in peptide 129–147, 2P or 3P in peptide 148–184 and may result in 10P and 11P isoforms. The identified fragment of κ-casein showed 4P to 5P variation by the level of phosphorylation of peptides 23–40 at 2P or 3P and 52–65/66 at 1P.

**Table 2:** Identification of phosphopeptides of caprine αS1, αS2, β, κ caseins and α-lactalbumin and β-lactoglobulin by nano-LC-MS/MS.

Protein name	Peptide no.	Peptide sequence*	Level of phosphorylation	Master protein accessions	Positions in master proteins	# Protein groups	Proteins identified	# PSMs	# Missed cleavages
αS1-casein	1	HPINHQGL**S**PEVLNENLLR	1P	Q8MIH4	[19–37]	1	8	1	0
	2	DIGSE**S**TEDQAMEDAK	1P	Q8MIH4	[58–73]	3	13	2	0
	3	DIG**S**E**ST**EDQAMEDAK	3P	Q8MIH4	[58–73]	2	13	3	0
	4	AG**SSSSS**EEIVPNSAEQK	5P	A0A0P0EL46	[76–93]	1	2	5	0
	5	**Y**IQKEDVPSER	1P	Q8MIH4	[95–105]	3	11	9	1
	6	**Y**LGYLEQLLR	1P	Q8MIH4	[106–115]	2	8	2	0
	7	KYNVPQLEIVPK**S**AEEQLHSMK	1P	Q8MIH4	[118–139]	3	5	2	2
	8	YNVPQLEIVPK**S**AEEQLHSMK	1P	Q8MIH4	[119–139]	3	5	1	1
	9	**S**AEEQLHSMK	1P	A0A0P0EL46	[129–138]	1	7	1	0
	10	**S**AEEQLHSMK	1P	Q8MIH4	[130–139]	1	7	4	0
αS2-casein	1	MEHVS**SS**EEPINIFQEIYK	2P	P33049	[19–37]	1	1	2	0
	2	MEHVS**S**SEEPINIFQEIYKQEK	1P	P33049	[19–40]	1	1	2	1
	3	MEHV**SSS**EEPINIFQEIYKQEK	3P	P33049	[19–40]	1	1	3	1
	4	EQLST**S**EENSKK	1P	P33049	[142–153]	1	2	2	1
	5	EQL**S**TSEENSKK	1P	P33049	[142–153]	1	2	1	1
	6	EQL**STS**EENSKK	3P	P33049	[142–153]	1	2	3	1
	7	TIDME**ST**EVFTK	2P	P33049	[154–165]	1	2	2	0
	8	TIDME**S**TEVFTK	1P	P33049	[154–165]	1	2	1	0
	9	TIDME**S**TEVFTKK	1P	P33049	[154–166]	1	2	2	1
	10	TIDME**ST**EVFTKK	2P	P33049	[154–166]	1	2	10	1
β-casein	1	IEKFQ**S**EEQQQTEDELQDK	1P	Q95L76	[45–63]	1	4	1	1
	2	FQ**S**EEQQQTEDELQDK	1P	Q95L76	[48–63]	1	4	4	0
	3	**Y**PVEPFTESQSLTLTDVEK	1P	Q95L76	[129–147]	1	8	1	0
	4	**Y**PVEPF**T**E**S**QSL**T**L**TD**VEK	6P	Q95L76	[129–147]	1	8	9	0
	5	**Y**PVEPF**T**E**S**Q**S**L**T**L**T**DVEK	6P	P33048	[129–147]	2	8	22	0
	6	LHLPLPLVQSWMHQPPQPL**S**P**T**VMFPPQSVLSLSQPK	2P	P33048	[148–184]	2	8	6	0
	7	LHLPLPLVQ**S**WMHQPPQPL**S**P**T**VMFPPQSVLSLSQPK	3P	P33048	[148–184]	2	8	9	0
	8	LHLPLPLVQSWMHQPPQPL**S**P**T**VMFPPQSVLSLSQPK	2P	Q95L76	[148–184]	1	8	3	0
	9	**D**MPIQAFLLYQEPVLGPVR	1P	Q95L76	[197–215]	1	4	2	0
	10	**D**MPIQAFLLYQEPVLGPVR	1P	P33048	[197–215]	2	4	1	0
κ-casein	1	**S**PAQ**T**LQWQVLPNTVPAK	2P	B2Z896	[23–40]	4	45	3	0
	2	**S**PAQ**T**LQWQVLPN**T**VPAK	3P	B2Z896	[23–40]	6	45	15	0
	3	HPHPHL**S**FMAIPPK	1P	B2Z896	[52–65]	4	48	1	0
	4	HPHPHL**S**FMAIPPKK	1P	B2Z896	[52–66]	4	48	1	1
	5	**Y**IPIQYVLSR	1P	Q7YRX4	[16–25]	2	26	2	0
β-lactoglobulin	1	GLDIQKVAGTW**YS**LAMAASDISLLDAQSAPLR	2P	P02756	[27–68]	1	2	2	1
	2	VAG**T**W**YS**LAMAA**SD**I**S**LLDAQSAPLR	6P	P02756	[33–58]	1	3	34	0
	3	VAG**T**W**YS**LAMAA**SD**ISLLDAQSAPLR	5P	P02756	[33–58]	1	3	14	0
	4	VAG**T**WYSLAMAA**SD**I**S**LLDAQSAPLR	4P	P02756	[33–58]	1	3	4	0
	5	VAG**T**W**YS**LAMAASDISLLDAQSAPLR	3P	P02756	[33–58]	1	3	8	0
	6	VAG**T**W**YS**LAMAA**S**DISLLDAQSAPLR	4P	P02756	[33–58]	1	3	2	0
	7	V**Y**VEELKP**T**PEGNLEILLQK	2P	P02756	[59–78]	1	3	5	0
	8	VYVEELKP**T**PEGNLEILLQK	1P	P02756	[59–78]	1	3	1	0
	9	IIAEK**T**KIPAVFK	1P	P02756	[89–101]	2	3	7	2
	10	**T**KIPAVFK	1P	P02756	[94–101]	1	3	2	1
	11	VLVL**DT**DYKK	2P	P02756	[110–119]	1	3	2	1
	12	**T**PEVDKEALEK	1P	P02756	[143–153]	1	3	2	1
	13	**T**PEV**D**KEALEK	2P	P02756	[143–153]	1	3	4	1
	14	**T**PEV**D**KEALEKFDK	2P	P02756	[143–156]	1	3	7	2
	15	TPEV**D**KEALEKFDK	1P	P02756	[143–156]	1	3	3	2
α-lactalbumin	1	IWCK**DD**QNPHSR	2P	B2YKX6	[78–89]	2	3	6	1
	2	IWCK**D**DQNPHSR	1P	B2YKX6	[78–89]	3	3	5	1
	3	KIL**D**KVGINYWLAHK	1P	P00712	[113–127]	1	1	4	2
	4	IL**D**KVGINYWLAHK	1P	P00712	[114–127]	1	1	7	1
	5	VGIN**Y**WLAHK	1P	P00712	[118–127]	1	1	3	0

^*^Phosphorylated amino acids are in bold and underlined.

Similarly, *LALBA* peptide 78–89 showed 2P and 1P variation at D_82_, D_83;_ peptide 113–127, 114–127 showed 1P at D_116_ and peptide 118–127 showed 1P at Y_122_ resulting in one more possible isoform up to 4P. In *BLG* the highest level of variation in phosphorylation was observed in peptide 33–58 with 3P, 4P, 5P and 6P at sites T_36_, Y_38_, S_39_, S_45_, D_46_ and S_48_. Furthermore, the peptides 59–78, 143–153/156 showed 1P and 2P variations resulting in 13P isoform of *BLG*. These findings indicated the different levels of isoforms in phosphorylation of the caprine milk proteins.

#### Phosphorylation of low abundance proteins

The sensitivity for the identification of phosphorylation sites in low abundance proteins was enhanced by combining 2DE and nLC-MS/MS approach. Phosphorylation was identified in 45 low abundant proteins and presented in Table 3. CREB binding protein (5P), inter-alpha-trypsin inhibitor heavy chain *H2* (2P), olfactory receptor *OR51D1* (3P), *GYCAM1* (1P), putative transcription factor (2P), Proteoglycan 4 (8P), and other proteins with varying level of phosphorylation were identified. Antiviral interferon *tau BB4* found only in goat milk was also detected with 7P level of phosphorylation.

**Table 3:** Phosphorylation in low abundance proteins of goat milk identified in the reference database with accession number and gene name.

Accession number	Protein name	Identified phosphosites	Gene name
A5JSS7	Glycosylation-dependent cell adhesion molecule-1	S87	GLYCAM1
G0Z386	Insulin-like growth factor binding protein-3 (Fragment)	S10, S13	IGFBP3
B7S4L4	Interferon tau BB4	S96; S97; D101; T102; T103; D117; D118	INTERFERONAB
Q7YS14	LDHA protein (Fragment)	S8, D9	LDHA
D2KMJ2	NADH-ubiquinone oxidoreductase chain 5	D297	ND5
A0A0C5B361	NADH-ubiquinone oxidoreductase chain 6	T95; Y105; Y106	ND6
G1DGB8	Putative transcription factor Ovo-like 1	T149, Y150	OVOL1
C6KGS6	Superoxide dismutase (Fragment)	T134, S143	MnSOD
W5P1Z9	Family with sequence similarity 222 member B	S111	FAM222B
W5P2D4	Neurofilament heavy	S62	NEFH
W5P440	HECT and RLD domain containing E3 ubiquitin protein ligase 4	S95/96/104	HERC4
W5P9A9	Olfactory receptor	T281; S282; Y292	OR51D1
W5PGE4	ATP/GTP binding protein 1	S780; S792	AGTPBP1
W5PHS2	Lipocln_cytosolic_FA-bd_dom domain-containing protein	T34	LCN2
W5Q0T0	Uncharacterized protein	S123	
W5Q219	Peptidase S1 domain-containing protein	S179	
W5Q2G4	Voltage-dependent calcium channel gamma-5 subunit	T102	CACNG5
W5Q9K7	Uncharacterized protein	T239	LOC101110727
W5QFN4	Succinyl-CoA:3-ketoacid-coenzyme A transferase	S507	OXCT1
A7MB45	Acyl-CoA synthetase short-chain family member 3, mitochondrial	S20; S24; S25	ACSS3
E1BDU1	Olfactory receptor	Y120	OR2K2
E1BDZ8	Enhancer of polycom+B63b homolog	T551	EPC2
F1MD32	CREB binding protein	Y226; T228; S247; S252; T258	CREBBP
F1MNW4	Inter-alpha-trypsin inhibitor heavy chain H2	S543; S549	ITIH2
F1MVC0	Uncharacterized protein	T201	CAD
G3X6Y3	Mediator of RNA polymerase II transcription subunit 14	Y1097; T1098; S1101	MED14
P00760	Cationic trypsin	S125; S127; S134; T130; S173; S175; T180; S181; S215	PRSS1
Q1JQA8	LysM and putative peptidoglycan-binding domain-containing protein 2	S21	LYSMD2
O00443	Phosphatidylinositol 4-phosphate 3-kinase C2 domain-containing subunit alpha	T927; Y928; S929; Y938; D947; S948; D952	PIK3C2A
O94986	Centrosomal protein of 152 kDa	S329; T331; T332; S338; D345; S349; S351	CEP152
P23471	Receptor-type tyrosine-protein phosphatase zeta	S1444; S1446; S1447; Y1448	PTPRZ1
P48664	Excitatory amino acid transporter 4	S562	SLC1A6
Q13753	Laminin subunit gamma-2	D498; Y500; D503; D524; S526; S528; D532	LAMC2
Q5JTW2	Centrosomal protein of 78 kDa	S117; S119; S120	CEP78
Q7Z5A7	Protein FAM19A5	T78; T79; D87	FAM19A5
Q8IXQ8	PDZ domain-containing protein 9	S235; S236; S238; S239; S241	PDZD9
Q8IY18	Structural maintenance of chromosomes protein 5	T3; S5; T8; S9; T10; S12; S16	SMC5
Q8N283	Ankyrin repeat domain-containing protein 35	S349; S355	ANKRD35
Q8N3J5	Protein phosphatase 1K, mitochondrial	Y352; S355; S360; S362	PPM1K
Q8N3Z6	Zinc finger CCHC domain-containing protein 7	S411	ZCCHC7
Q8WU58	Protein FAM222B	S111; D115; D117; T119	FAM222B
Q92954	Proteoglycan 4	Y38; S39; D41; T43; D47; Y48; Y53; D59	PRG4
Q96SR6	Zinc finger protein 382	S533; T541; T546; T547	ZNF382
Q9BXX0	EMILIN-2	D544; D551; D573	EMILIN2
Q9H251	Cadherin-23	T259; D262; D264	CDH23

#### Isoform in different breeds

The identified 123 common UniprotKB ID proteins with varying level of phosphorylation across the 10 breeds were observed by comparing databases. The phosphosites and phosphoproteins observed in each breed are presented in Table S3. *CSN1S1* (Q8MIH4)9P (S_27_, D_58_, S_61_, S_63_, T_64_, D_66_, Y_95_, Y_106_, S_130_) was identified in samples of Himalayan local goats, whereas, D_100_, S_138_ phosphosites were observed in Osmanabadi goat. Similarly, *CSN1S2* (P33049) 10P (S_23_, S_24_, S_25_, S_145_, T_146_, S_147_, T_154_, S_159_, S_160_, T_161_) was observed in Jamunapari goat. Phosphorylation at (S_151_, D_156_) and T_164_ was observed in Himalayan local goat and Osmanabadi goat, respectively. *CSN2* (Q95L76) showed 13P (S_50_, D_62_, Y_129_, T_135_, S_137_, T_141_, T_143_, D_144_, S_157_, S_167_, T_169_, S_181_, D_197_) in Jakhrana goats. *CSN3* (Q7YRX4) 5P (Y_16_, S_24_, S_60_, T_64_, S_95_) was observed in Attapady Black goats. α-lactalbumin (P00712) exhibited 4P (D_82_, D_83_, D_116_, Y_122_) in Himalayan local goat and β-lactoglobulin (P02756) showed presence of 16P (D_29_, T_36_, Y_38_, S_39_, S_45_, D_46_, Y_60_, T_67_, T_94_, D_114_, T_115_, D_116_, Y_117_, T_143_, D_147_, D_155_) in Osmanabadi, 15P in Attapady Black, 13P in Gaddi goats and sites S_48_, D_51_ and S_54_ were also noted.

Similarly, the low abundant proteins were identified in different breeds with varying sites of phosphorylation. Proteins Proteoglycan 4 (PRG4) showed 8P isoform in Barbari, *PIK3C2A*, *CEP152* observed 7P each and SLC1A6 1P in Barbari. Interferon *tau BB4* (7P), *CREBBP* (5P), Cationic trypsin (8P) isoforms were observed in Jamunapari. *PDZD9* and *SMC5* observed exhibited 5P and 7P in Osmanabadi goats. Emilin-2 (3P), *FAM222B* (4P), *MnSOD* and *OVOL1* each with 2P isoforms were observed in Sirohi goats.

#### Other post translational modifications

Other post translational modification such as oxidation and carbamidomethylation were also observed and presented in Table 4. Caseins showed only oxidation and phosphorylation. Peptides of *LALBA* and *BLG* showed both oxidation and carboxymethylation other than phosphorylation. β-casein showed carboxymethylation on 7 sites (M_117_, M_124_, M_159_, M_160_, M_171_, M_172_, M_198_) whereas; α-S2 casein displayed 3 oxidation sites M_42_, M_157,_ M_206_. PTM in proteins α-lactalbumin were observed as C_80_, C_110_, C_130_, C_139_ and C_84_, C_178_, M_42_, M_163_ on β-lactoglobulin. Low abundant proteins also exhibited oxidation and carboxymethylation such as interferon *tau BB4* (C_122_, M_127_), laminin subunit *LAMC2* (C_493_, C_496_, C_514_, C_517_, C_519_, C_531_), olfactory receptor *OR2K2* (C_97_, C_112_, M_101_, M_107_, M_118_), *ND5* (C_279_, C_291_, M_277_), proteoglycan (C34; C44; C46; C50; C56; C57), keratin *KRT1* (C_49_, M_259_, M_262_, M_296_) and histone showed M_60_, M_63_ and/or C_111_ modifications.

**Table 4:** Oxidation and carboxymethylation sites as observed in high and low abundance goat milk proteins.

Modifications (oxidation, carboxymethylation)	Accession number	Protein description	Species	Gene name
C110, C130, C139	P00712	Alpha-lactalbumin	*Capra hircus*	LALBA
C122; M127	B7S4L4	Interferon tau BB4	*Capra hircus*	INTERFERONAB
C125	Q6S4N9	Fatty acid binding protein 3	*Capra hircus*	FABP3
C148, C151	G1DGB8	Putative transcription factor Ovo-like 1	*Capra hircus*	OVOL1
C279; C291; M277	D2KMJ2	NADH-ubiquinone oxidoreductase chain 5	*Capra hircus*	ND5
C42, M60, M97	B2Z896	Kappa casein	*Capra hircus*	CSN3
C494, M84/86	C6KJ77	Breast cancer resistance protein	*Capra hircus*	ABCG2
C80	A0A0M4RF90	Chemokine receptor 2	*Capra hircus*	CCR2
C80, C110	B2YKX6	Alpha-lactalbumin	*Capra hircus*	LALBA
C84; C178; M42; M163	P02756	Beta-lactoglobulin	*Capra hircus*	LGB
C9	H2ETE9	Natriuretic peptide C	*Capra hircus*	NPPC
M11	G0Z386	Insulin-like growth factor binding protein-3 (Fragment)	*Capra hircus*	IGFBP3
M117, M124, M159, M171, M198	Q95L76	Beta-casein	*Capra hircus*	CSN2
M117, M124, M198, M159, M160, M171, M172	P33048	Beta-casein	*Capra hircus*	CSN2
M150, M138	Q8MIH4	Alpha s1 casein	*Capra hircus*	CSN1S1
M276	A9UFM4	Hormone sensitive lipase (Fragment)	*Capra hircus*	HSL
M33	C6ZP43	I alpha globin	*Capra hircus*	HBA1
M36,	A0A2U8URJ6	Beta-casein (Fragment)	*Capra hircus*	CSN2
M36; M48; M75	Q5YD57	Beta-casein	*Capra hircus*	CSN2
M42, M157; M206	P33049	Alpha-S2-casein	*Capra hircus*	CSN1S2
M54, M70	I1X3V0	Promyelocytic leukemia zinc finger protein (Fragment)	*Capra hircus*	PLZF
M60, M63	G1DFR8	Histone H2B type 3-A	*Capra hircus*	HIST3H2BA
M88	Q6R649	Keratin, type I cytoskeletal 27	*Capra hircus*	KRT27
M90	Q7YRX4	Kappa-casein (Fragment)	*Capra hircus*	CSN3
M99, M103	A0A0C5B361	NADH-ubiquinone oxidoreductase chain 6	*Capra hircus*	ND6
C186, C189	Q0ZBS4	Pol protein (Fragment)	*Caprine arthritis encephalitis virus*	POL
M459	Q9DKV8	Pol protein	*Caprine arthritis encephalitis virus*	POL
C111	E1BGN3	Histone H3	*Bos taurus*	HIST2H3D
C125	P10790	Fatty acid-binding protein, heart	*Bos taurus*	FABP3
C156	F1MCF8	Ig-like domain-containing protein	*Bos taurus*	HAVCR2
C176; M161; M40; C82; C122; C135; C137;	G5E5H7	Deleted entry	*Bos taurus*	LOC615237
C183	Q7YS80	Myogenic factor 6	*Bos taurus*	MYF6
C30; C48; C64; C132; C139; C160; C171; C185; C196; C206; C220; C233; M109; M183	P00760	Cationic trypsin	*Bos taurus*	PRSS1
C47; C130; C139	P00711	Alpha-lactalbumin	*Bos taurus*	LALBA
C97; C112; M101; M107; M118	E1BDU1	Olfactory receptor	*Bos taurus*	OR2K2
M124; M200	P02666	Beta-casein	*Bos taurus*	CSN2
M125	F1MC11	Keratin, type I cytoskeletal 14	*Bos taurus*	KRT14
M127	P02668	Kappa-casein	*Bos taurus*	CSN3
M196	F1MHA3	Ig-like domain-containing protein	*Bos taurus*	TARP
M562	F1MNW4	Inter-alpha-trypsin inhibitor heavy chain H2	*Bos taurus*	ITIH2
M60; M63	E1B8G9	Histone H2B	*Bos taurus*	HIST3H2BB
M93	F1MQL3	Peptidase S1 domain-containing protein	*Bos taurus*	LOC615237
Acetylation	Q8IY18	Structural maintenance of chromosomes protein 5	*Homo sapiens*	SMC5
C111	Q71DI3	Histone H3.2	*Homo sapiens*	HIST2H3A
C115	Q5JTW2	Centrosomal protein of 78 kDa	*Homo sapiens*	CEP78
C1442; C1445	P23471	Receptor-type tyrosine-protein phosphatase zeta	*Homo sapiens*	PTPRZ1
C249	Q8IXQ8	PDZ domain-containing protein 9	*Homo sapiens*	PDZD9
C34; C44; C46; C50; C56; C57	Q92954	Proteoglycan 4	*Homo sapiens*	PRG4
C49; M259; M262; M296	P04264	Keratin, type II cytoskeletal 1	*Homo sapiens*	KRT1
C493; C496; C514; C517; C519; C531	Q13753	Laminin subunit gamma-2	*Homo sapiens*	LAMC2
C553; M564	Q9BXX0	EMILIN-2	*Homo sapiens*	EMILIN2
C77	P02538	Keratin, type II cytoskeletal 6A	*Homo sapiens*	KRT6A
C77	P04259	Keratin, type II cytoskeletal 6B	*Homo sapiens*	KRT6B
C85	Q7Z5A7	Protein FAM19A5	*Homo sapiens*	FAM19A5
M109	P07477	Trypsin-1	*Homo sapiens*	PRSS1
M119	P02533	Keratin, type I cytoskeletal 14	*Homo sapiens*	KRT14
M150; M271; M306	P13645	Keratin, type I cytoskeletal 10	*Homo sapiens*	KRT10
M157; M234; M245; M324; C406	P35527	Keratin, type I cytoskeletal 9	*Homo sapiens*	KRT9
M16	Q8NCI6	Beta-galactosidase-1-like protein 3	*Homo sapiens*	GLB1L3
M2243	Q96RW7	Hemicentin-1	*Homo sapiens*	HMCN1
M334	O94986	Centrosomal protein of 152 kDa	*Homo sapiens*	CEP152
M406	Q13873	Bone morphogenetic protein receptor type-2	*Homo sapiens*	BMPR2
M60; M63	O60814	Histone H2B type 1-K	*Homo sapiens*	HIST1H2BK
C66, C72	F1AWZ7	PP107	*Orf virus*	
Acetylation	W5Q9P7	Cofilin-1	*Ovis aries*	CFL1
C115	W5PS91	Histone H3	*Ovis aries*	H3FA
C30; C39; C47; C80;C110; C130; C139	W5QD52	Alpha-lactalbumin	*Ovis aries*	LALBA
C708	W5P440	HECT and RLD domain containing E3 ubiquitin protein ligase 4	*Ovis aries*	HERC4
C811; M790	W5PGE4	ATP/GTP binding protein 1	*Ovis aries*	AGTPBP1
M102	W5PXK1	Peptidase S1 domain-containing protein	*Ovis aries*	PRSS1
M102; M115; M189; C36; C54; C70; C177; C191	W5Q219	Peptidase S1 domain-containing protein	*Ovis aries*	PRSS1
M124; M159; M198	P11839	Beta-casein	*Ovis aries*	CSN2
M125; M199	W5PLC2	Beta-casein	*Ovis aries*	CSN2
M127	A0A059T9N6	Kappa-casein	*Ovis aries*	CSN3
M138; M150	D2D3I8	Alpha s1 casein	*Ovis aries*	CSN1S1
M157; M206	E7BQS1	Alpha-S2-casein	*Ovis aries*	CSN1S2
M163; C84; C178	P67976	Beta-lactoglobulin	*Ovis aries*	BLG
M22	R4R2H5	Beta-caesin	*Ovis aries*	CSN2
M288	W5P9A9	Olfactory receptor	*Ovis aries*	OR51D1
M408	W5Q885	Receptor protein serine/threonine kinase	*Ovis aries*	BMPR2
M47; M80; M130; M139	P09462	Alpha-lactalbumin	*Ovis aries*	LALBA
M518; C502	W5QFN4	Succinyl-CoA:3-ketoacid-coenzyme A transferase	*Ovis aries*	OXCT1
M60; M63	W5QA24	Histone H2B	*Ovis aries*	HIST2H2BE
M84	W5Q6L8	IF rod domain-containing protein	*Ovis aries*	VIM
M479	A0A088DBX4	Haemagglutinin protein	*Peste-des-petits-ruminants virus*	H

### Functional prediction and protein interaction analysis and bioactive peptides

The identified proteome was classified into functional categories such as biological process (BP) (87.8% genes), cellular component (CC) (97.6% genes) and molecular function (MF) (92.7% genes) using DAVID 6.8. The detailed annotations including UP-Keywords from DAVID software are available in Table S4. Enriched GO terms for BP included single-multicellular organismal process (*P* value 3.63 E−08), cellular component organization (*P* value 9.51 E−10), response to stress (*P* value 1.27 E−07), anatomical structure development (*P* value 2.94 E−07), defense process (*P* value 4.99 E−07), response to hypoxia (*P* value 2.57 E−04). The proteins mostly localised in membrane bound organelle (*P* value 1.72 E−06), and extracellular region (*P* value 4.05 E−46) and extracellular exosome (*P* value 1.27 E−51). The molecular functions (MF) largely involved binding activity such as protein binding, DNA binding, nucleosome binding, anion binding, antioxidant activity (*P* value 2.94 E−04) and structural molecular activities (*P* value 2.51E−39) were also observed. The UP_Keywords comprised antimicrobials, phosphoprotein, disease mutation and methylation.

**Figure 1:**
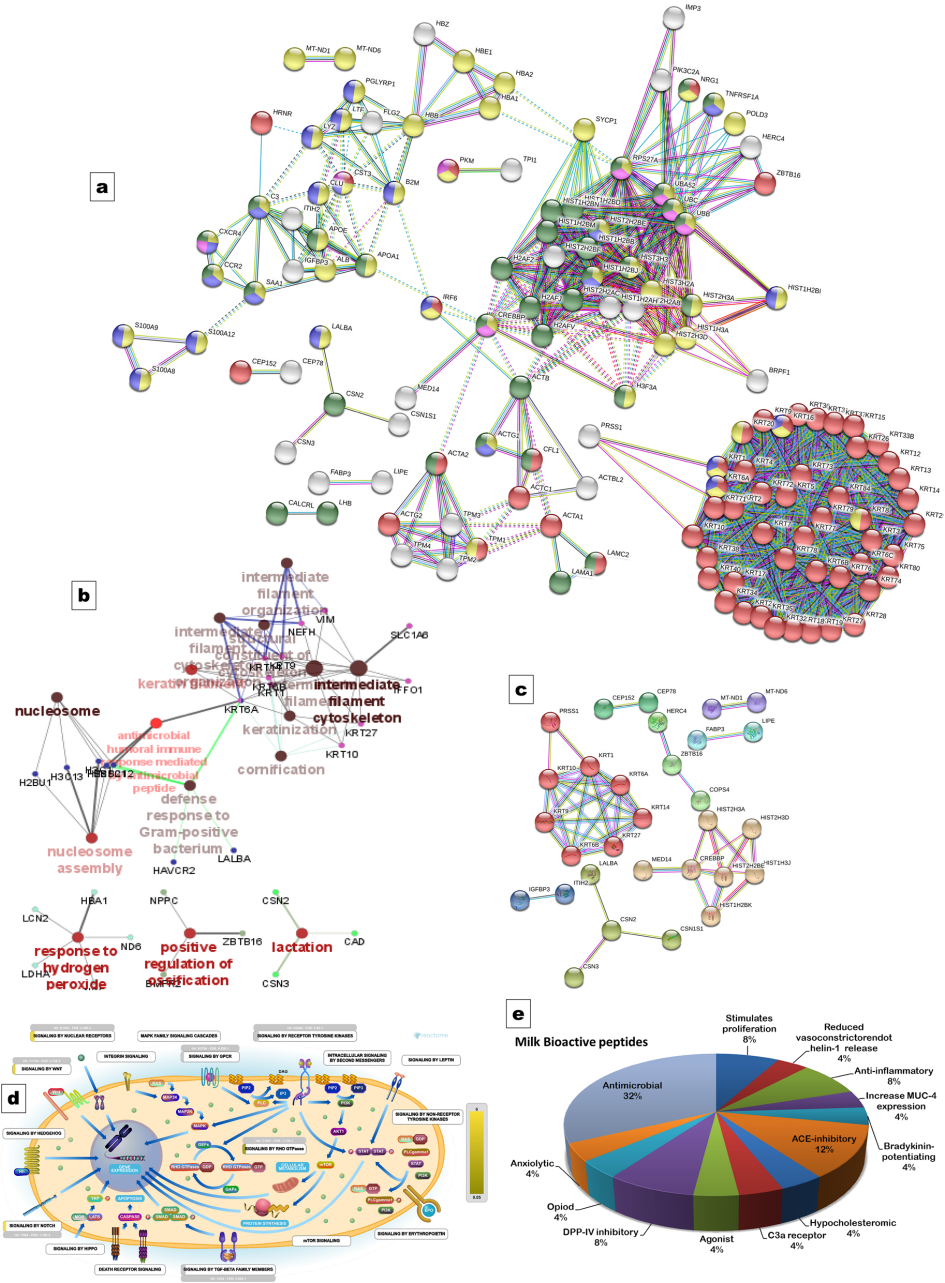
Functional prediction and protein-protein interaction analysis: MCL clustering by STRING for all the identified proteins (**a**); Cytoscape network analysis of PTM gene subset (**b**); MCL clustering by STRING for PTM protein subset (**c**); Signal transduction pathway from reactome database for PTM protein subset (**d**); functional attributes of identified bioactive peptides (**e**).

The interaction of proteins was analysed by STRING by MCL clustering of the identified proteome categorised the proteins in 16 clusters at highest confidence (>90%) score as depicted in Figure 1a. The network comprised of three major groups where keratin proteins shared distinct close interconnected cluster. Proteins found in defense response were also involved in response to stress, signal transduction and tissue development.

#### Functional analysis of PTM proteins

The GO functional annotations for PTM genes subset were enriched using DAVID 6.8 for classification into BP (87.5% genes), CC (94.4% genes) and MF (87.5% genes). The details of annotations are presented in Table S5. Enriched GO terms included developmental process (*P* value 0.02069), response to stress (*P* value 0.0099), response to drug (*P* value 0.0040), biogenesis (*P* value 0.048), structural molecular activity (*P* value 5.19 E−04), protein dimerization activity (*P* value 0.0092) and extracellular space (*P* value 3.00 E−06).

The phosphoprotein subset when analysed in Cytoscape Cluego identified 76 genes to generate a network with 6 functional groups at different levels. The details of annotations are presented in Table S6, Supplementary data file 1. The categorized functional groups were lactation, response to hydrogen peroxide, positive regulation of ossification, defense response to gram-positive bacterium, cornification and Systemic lupus erythematosus. Keratin proteins were associated with cornification, *H2BC12*, *H2BC21* and *KRT6A* with antimicrobial humoral immune response mediated by antimicrobial peptide. Proteins *BMPR2*, *NPPC*, ZBTB16 found in defense response to gram-positive bacteria, *CAD*, *CSN2*, *CSN3* in lactation and neurofilament in structural constituent of cytoskeleton. Estrogen signaling pathway was detected and proteins *H3C1, H3C13, H3C15* were associated with pathways such as alcoholism, histone modifications, systemic lupus erythematosus (Fig. 1b).

The PTM gene subset exhibited a network comprising of 8 clusters in STRING (Fig. 1c) The keratin proteins clustered with *PRSS1* (Trypsin); *CSN2*, *CSN1S1*, *CSN3* and *LALBA* were identified in single cluster and histone proteins interacted closely with CREB-binding protein.

The identifiers (PTM genes) were analysed in Reactome database, where 61 identifiers were found in 332 pathways hit by at least one of them. The majority of proteins were associated with signal transduction, immune system, developmental biology and metabolism pathways. Pathways such as signaling by nuclear receptors, WNT, Notch and RHO GTPase were significant with *p* < 0.05 (Fig. 1d). The identified 17 phosphoproteins involved in signal transduction pathways included *BMPR2, CCR2, CFL1, CREBBP, CSN2, H3FA, HIST1H2BK, HIST2H2BE, HIST2H3A, HIST2H3D, HIST3H2BB, LAMC2, OR2K2*, *OR51D1* and *PRG4*. The details of pathways has been given in Table S7, Supplementary data file 1.

#### Identification of bioactive peptides

The 201 peptide sequences with identified PTM sites were grouped as long (>25 amino acids), medium (7–25 amino acids) and small (<7 amino acids). The identified phosphopeptides were categorized as long (62 peptides), medium (145 peptides), and small (1 peptide). The functions of these peptides were determined from MBPDB for 80% alignment with the known peptides are presented in Table 5. αS1- casein peptide 106–115 was anxiolytic and peptide 107–116 was antimicrobial. κ-casein peptide 46–55 exhibit C3a receptors agonist and opioid functions. β-lactoglobulin peptide 158–164 and β-casein 123–128 showed multiple functions. β-casein 199–217 was anti-inflammatory and α-lactalbumin 118–127 ACE inhibitory. The identified bioactive peptides exhibited anti-microbial activity, DPP-IV inhibitory, anti-inflammatory, ACE inhibitory, antioxidant, proliferating, anti-oxidative, opioid, anti-hypertensive, anxiolytic and hypocholesterolemic functions (Fig. 1e).

**Table 5:** The function of identified bioactive peptide from MBPD.

Search peptide	Protein ID	Peptide	Protein description	Position in protein	Function	% alignment	e-value	Alignment length	mismatches
ALKALPMHIR	P02754	ALKALPMHIR	β-lactoglobulin	155–164	stimulates proliferation	100	1.63E−10	10	0
ALPMHIR	P02754	ALPMHIR	β-lactoglobulin	158–164	stimulates proliferation, Reduced vasoconstrictorendothelin-1 release, ACE-inhibitory	100	8.50 E−07	7	0
AMKPWTQPK	P02663	AMKPWIQPK	α-S2-casein	204–212	ACE-inhibitory	88.89	7.75 E−07	9	1
DMPIQAFLLYQEPVIGPVR	P02666	DMPIQAFLLYQEPVLGPVR	β-casein	199–217	Anti-inflammatory	94.73	3.33E−19	19	1
DMPIQAFLLYQEPVLGPVR	P02666	DMPIQAFLLYQEPVLGPVR	β-casein	199–217	Anti-inflammatory	100	1.39E−21	19	0
EMPFPK	P02666	EMPFPK	β-casein	123–128	ACE-inhibitory, Increase MUC4 expression, Bradykinin-potentiating, Antimicrobial	100	1.49 E−05	6	0
HKEMPFPK	P02666	HKEMPFPK	β-casein	121–128	Antimicrobial	100	4.88 E−08	8	0
IIAEKTKIPAVFK	P02756	IIAEKTKIPAVF	β-lactoglobulin	89–100	Antimicrobial	92.30	8.33E−13	12	0
TPEVDKEALEK	P02754	TPEVDDEALEK	β-lactoglobulin	141–151	DPP-IV Inhibitory, Antimicrobial	90.91	2.50 E−09	11	1
TPEVDKEALEK	P02756	TPEVDKEALE	β-lactoglobulin	143–152	Antimicrobial	90.91	2.49E−10	10	0
VGINYWLAHK	P00711	VGINYWLAHK	α-lactalbumin	118–127	ACE-inhibitory	100	1.63E−10	10	0
VKETMVPK	P33048	KETMVPK	β-casein	114–120	Antimicrobial	87.5	1.35 E−06	7	0
VLVLDTDYKK	P02754	VLVLDTDYK	β-lactoglobulin	108–116	DPP-IV Inhibitory, Antimicrobial	90	4.34 E−09	9	0
VYVEELKPTPEGNLEILLQK	P02754	VYVEELKPTPEGDLEILLQK	β-lactoglobulin	57–76	Hypocholesterolemic	95	1.97E−20	20	1
YIPIQYVLSR	P02668	YIPIQYVLSR	κ-casein	46–55	C3a Receptors agonist, Opioid	100	1.63E−10	10	0
YLGYLEQLLR	P02662	YLGYLEQLLR	α-S1-casein	106–115	Anxiolytic	100	1.63E−10	10	0
YLGYLEQLLR	P02662	LGYLEQLLRL	α-S1-casein	107–116	Antimicrobial	90	6.03 E−09	9	0

## Discussion

Non-bovine milk is attracting the researcher’s attention due to its nutrition and therapeutic applications. Goat milk, is leading in the area for nutraceutical formulation and drug development using goat mammary gland as a bioreactor. Goat milk has unique chemical, biochemical, physical and nutritional characteristics and has higher digestibility and lower allergenicity over cow milk^25,26^. Post-translational modifications (PTMs) of milk proteins contributed to their biological functions and their compositional complexity^27^. It has been commonly observed that phosphorylation of casein occurs at S or T amino acid residues in tripeptide sequences S/T-X-A, where X represents any AA residue and A is an acidic residue^5^. In the present study, we have identified phosphorylation occurring in S, T, Y, D amino acids and other post translational modifications as carboxymethylation, oxidation and acetylation. The phosphosites on Asp residues have been reported for the first time in goat milk. Post translational modification of proteins plays an important role to regulate the cellular processes, protein function, protein localization, and formation of protein complex. In eukaryotic cells, protein phosphorylation is among the most frequent post translational modifications^28^. Post-translational modifications in proteins are crucial for the activity state, localization and protein-protein interactions^29^. Therefore, molecular diversity of goat milk proteins needs to be explored to identify post translational modifications for studying potential biological role and protein–protein interaction.

The present study focused on obtaining a comprehensive profile of the PTM sites of goat milk casein and non-casein protein and their interaction. Proteomics as a tool have been employed for the discovery, and characterization of post translational modifications such as phosphorylation, oxidation^30–32^. Electrospray ionization (ESI) mass spectrometry (MS) is suitable for studying PTM, including phosphorylation and glycosylation, since the technique provides molecular mass determination of native proteins. In this study, we analysed the goat milk proteins to identify both casein and non-casein PTM sites using nLC-MS/MS. We processed distinct protein spots by mass spectrometry for identification of phosphorylation, oxidation, acetylation and caramidomethylation. A peptidome of 201 peptide sequences with post translational modifications identified 86 proteins/120 UniprotKB accessions (Table S2). The phosphorylation site identified on the amino acids serine, threonine, tyrosine and aspartic acid was 128, 70, 42 and 47, respectively. Serine showed highest affinity for phosphate group in the present study and confirming earlier reports^18^.

Phosphorylation has been well characterized in the bovine caseins^33,34^. There are various reports targeting casein fractions in bovine and non-bovine milk by various proteomic approaches^32,35–39^. The majority of bovine caseins exist in a phosphorylated form, and the phosphorylated residues vary from individual variants possessing one phosphorylated residue (κ-CN) to 13P for others (αS2 CN)^40^. Bijl and others^41^ demonstrated that high αS1-CN-8P concentration in bovine milk is a great benefit for the production of uncooked curd cheese because αS1-CN-8P is hydrolyzed more efficiently by chymosin during ripening. Bovine proteome analysis showed more than 30 phosphorylated proteins which included 5 *CSN2*, 15 *CSN1S1*, 10 *CSN1S2* and 4 *CSN3* casein components^42^. Similarly, donkey milk showed 11 *CSN3*, 6 *CSN1S1* and 3 *CSN1S2* casein components^43^.

Goat milk proteins have been analysed using MS/MS^44–49^. The present study resulted in 105 phosphopeptides from casein and non-casein proteins from all the analysed samples. The conserved peptide sequence of (SSSEE) in casein was also observed in *CSN1S1* and *CSN1S2* at 1P, 2P and 3P. The phosphosites were identified in casein and whey proteins and on 45 other low abundance proteins. The total number of phosphosites observed in the major milk proteins associated with S, T, Y and D residues were 32, 18, 11 and 21, respectively. The phosphorylation sites observed for *CSN2, CSN1S1, CSN1S2,* and *CSN3* were 11P, 13P, 17P and 6P, respectively (Table 1). However, whey proteins *BLG* showed 19 phosphosites (7D, 3Y, 5T, 4S) and LALBA showed 4 phosphosites (3D, 1Y). The identified phosphopeptides resulted in 12P, 8P, 11P, 5P, 13P and 4P isoforms of *CSN1S1, CSN1S2, CSN2, CSN3, BLG* and *LALBA*, respectively. Beta casein showed highest degree of variation in phosphorylation with identification of 17P sites and other PTM such as oxidation in the identified peptides. A higher number of casein phosphopeptides and phosphorylation sites are reported in the present study in comparison to previous study^18^.

Casein and whey proteins are post translationally modified by proteolysis by the milk enzymes, formation of disulphide bond by oxidation of cysteine, differential phosphorylation levels of serine and threonine, and glycosylation of threonine residues^50^. The phosphorylation degree of αS-casein is a prime factor affecting the technological properties of milk. Therefore, “signature peptides” and “caseome” analysis are being used to investigate adulteration in milk of different species^51^. The identification of cheese from different species has been authenticated by proteolytic peptides^52,53^. Therefore, proteome analysis of fermented milk products should be carried out due to their nutritional and health economic importance.

The present study reported 45 non casein phospho proteins assigned to various metabolic pathways (Table 3). The identified PTM sites varied in milk samples of different goat breeds (Table 4). Identification of low abundance proteins in milk is difficult as single step analysis fails to detect a large proportion of these proteins. Moreover, to overcome the limited entries in the caprine database, other reference database were used for identification of low abundance proteins. The varying levels and sites of phosphorylation in different breeds may be attributable to various physiological or environmental conditions under the influence of different agro-climatic regions. The phosphopeptides assigned to these proteins were mainly mono- or bi-phosphorylated (Table 2).

The other post translational modifications such as acetylation, oxidation, and carbamidomethylation have also been reported (Table 4). The post translational modifications play an important role in protein/peptide functioning and their interaction. N-terminal acetylation increases peptide stability by preventing N-terminal degradation^54^. Peptides with carbamidomethylation are mainly used in peptide mass fingerprinting for identification and characterization of proteins^55^.

In the present study, the protein-protein interaction was analysed to know about the functional properties of proteins (Fig. 1). The GO annotations were analysed using DAVID, network and interactions using Cytoscape and STRING and pathways analysis using Reactome database. The keratin proteins interacted with trypsin (*PRSS1*) and histone proteins interacted with CREB binding protein (*CREBBP*). The identified phosphoproteins were associated with lactation, response to stress, histone modification, cornification and signaling pathways (Tables S5, S6, S7). It has been reported that caseins are associated with other secreted calcium (phosphate)–binding phosphoproteins, such as osteopontin, in milk^56^. Protein phosphorylation is vital for the regulation of metabolism, proliferation, inflammation, apoptosis, signaling and other important physiological processes. Autophosphorylation increases the catalytic efficiency of the receptor and provides binding sites for the assembly of downstream signaling complexes^57^. Caseins form micelles which vary from species to species, and when cleaved, generated bioactive peptides, having potential functions making them protein of interest. This gastrointestinal degradation may be the consequence of enzymatic hydrolysis, fermentation and other processes used in dairy production^58^.

The identification and characterization of phosphorylation sites are required to explore signaling networks of milk proteins. Phosphorylation-site provides definitive information on functional relationships between signaling proteins. The peptides released by enzymatic hydrolysis have specific biological functions due to their functional and interactions at cellular level^59^. The identified phospho bioactive peptides were mainly anti-microbial followed by ACE inhibitory, DPP-IV inhibitory, proliferating functions. Anti-oxidative, antioxidant, anxiolytic and hypocholesterolemic peptides were also confirmed from goat milk proteins (Fig. 1e). Non-bovine milk, for their health potential, economic value and the bioactive components/peptides, the milk protein fractions are being extensively investigated.

The goat milk protein genotypes have been observed in different breeds. Protein and casein content depend on allelic variants and breeds in different regions^60–63^. CSN1S1 gene acts as natural model and different genotypes occur due to interallelic combinations^64^. Indian goats have higher frequency of A and B alleles^65,66^. Sannen, Alpine and other European goat breeds have higher frequency of medium alleles like C, D, E, F^60,61,67,68^. Therefore, interallelic combination in casein complex leads to differential protein synthesis as well as other processing properties. Therefore, including different breeds/genotypes will definitely affect the proteome identification and post translational modifications pattern.

## Conclusion

Proteomic analysis has been carried out to study human, bovine and non-bovine milk for nutrition and therapeutic applications. Phosphorylation has been well characterized due to several technical, practical and bioinformatics approach. The present study identified 201 peptides showing post translational modifications in goat milk. The phosphorylation at Asp residues is reported first time in goat milk proteins. The rare conserved peptide sequence of (SSSEE) in casein was observed in casein phosphopeptides (CSN1S1, CSN1S2). Phosphorylation regulates protein functions, such as biological activity, interaction and stabilization by causing conformational changes in the protein. Therefore, the identification of the post-translational modifications of the milk proteome has become necessary and may provide newer insight to extend the milk proteome and its potential biological role.

## Materials and methods

**Figure 2:**
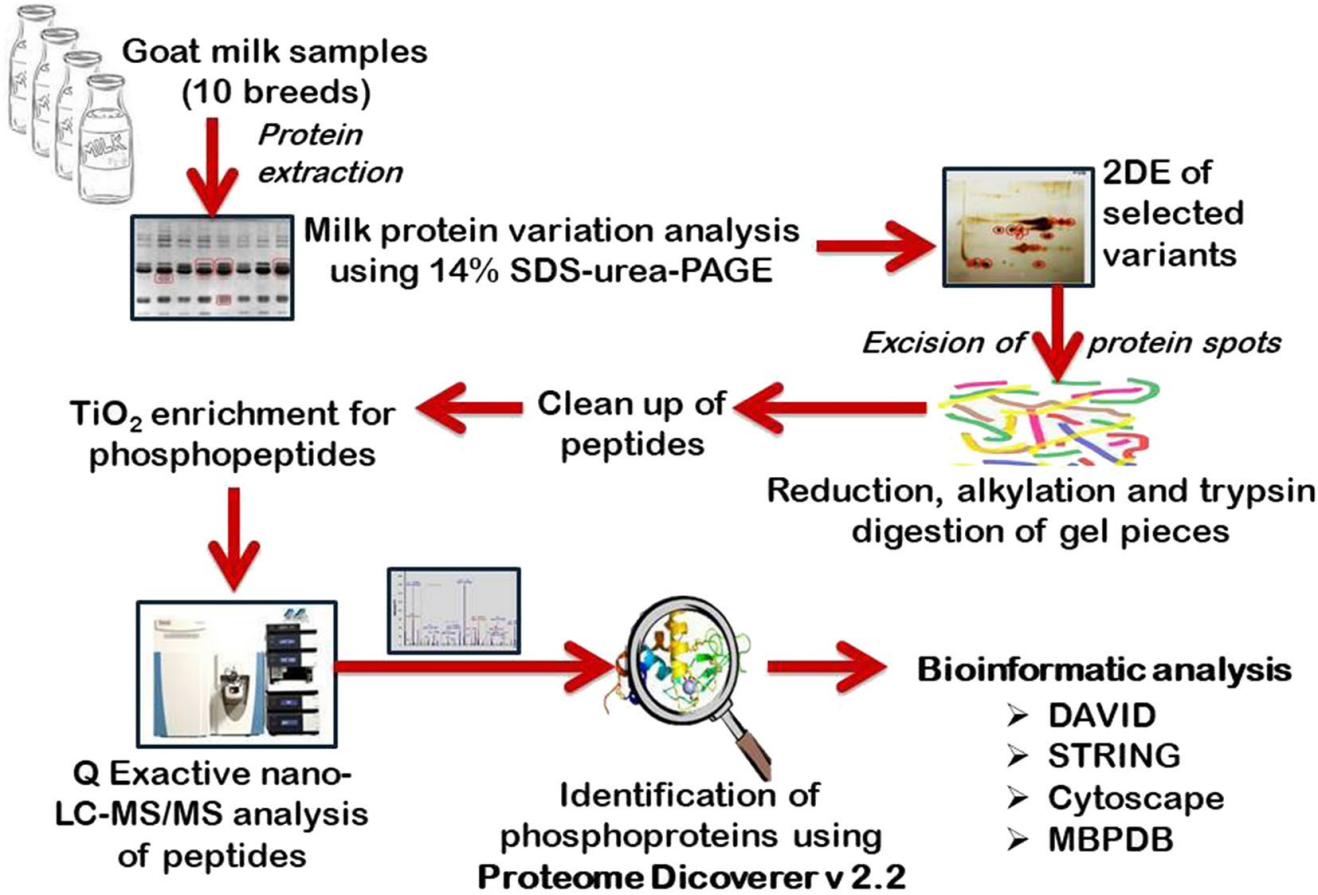
Flow diagram showing the methodology of PTM analysis.

The work flow of the present study has been depicted in Fig. 2.

### Milk collection and description of genetic stock

Goat milk samples were collected from 1240 animals belonging to 10 goat breeds/ genotypes during the postpartum days 30–65. Milk samples were collected from natural habitats of the breeds belonging to different geographical and agro-climatic regions of India. Samples were collected between 7:30 and 9:00 hrs. by hand milking. After disinfecting the udder, 30–40 ml of milk was collected directly to the collection tubes, transported to the laboratory at 4ºC within 24 hrs. and stored at -20ºC. Milk subsamples for protein analysis were stored at -40ºC until further analysis. All sample collection was conducted in accordance with institutional practice and the study was approved by Institutional animal ethics committee (IAEC).

**Figure 3:**
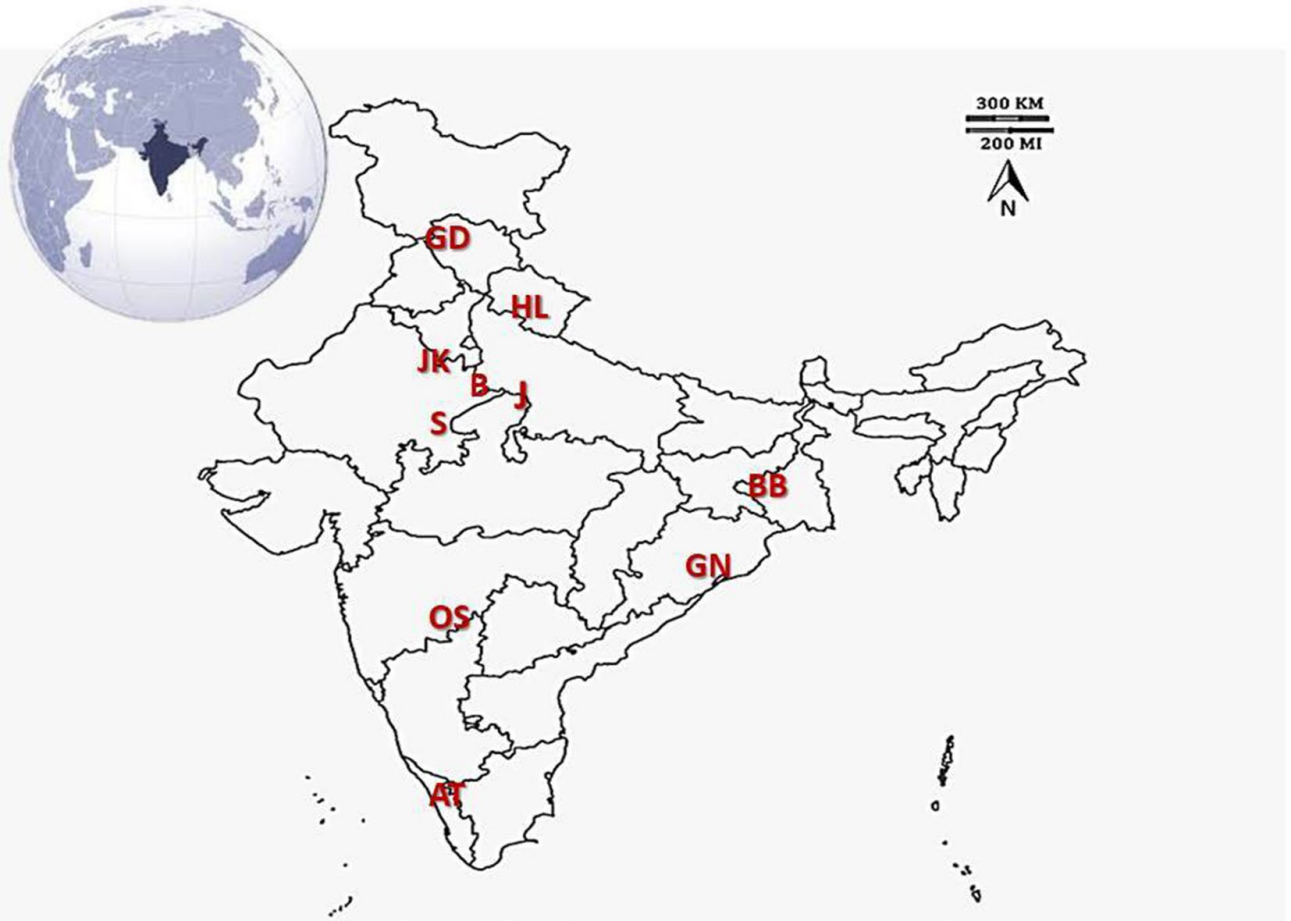
Geographical location of goat milk sampling. The breeds are abbreviated in the region of geographical origin. GD, Gaddi; HL, Himalayan local; JK, Jakhrana; B, Barbari; J, Jamunapari; S, Sirohi; BB, Black Bengal; GN, Ganjam; OS, Osmanabadi; AT, Attapady Black.

The breeds analysed in the study belonged to arid, semi-arid, humid, coastal and mountain regions with different grazing conditions (Fig. 3). The details of samples collected with the description of natural habitats from each breed are presented in Table S1, Supplementary data file 1. The animals were apparently healthy and the body condition score was satisfactory (3–4). The animals are mainly reared under semi-intensive system depending mostly on field grazing and supplementation of dry fodder, concentrate and mineral mixture.

### Gel based milk protein analysis

The milk protein variants were analysed by SDS-PAGE and details have been described elsewhere^44^. The skimmed milk samples were centrifuged at 12000g, 4ºC, 15 min to obtain clear transparent aqueous layer. This layer was separated, quantified and reduced in Laemmli’s sample buffer for analysis by SDS-PAGE. Milk proteins were resolved on 14% SDS and urea PAGE using 8 µg in each lane. The gels were stained with commasie brilliant blue (R250) and subsequently scanned in the Gel documentation system (Alpha Innotech Corporation, USA). Milk protein variants were identified by comparing the allelic variation with reference samples (confirmed by sequencing) by determining the molecular weight.

The protein variants (n=21) identified in the 10 breeds using SDS-PAGE were selected for analysis by 2DE. Individual protein samples were subjected to in–gel rehydration of IPG strips (SERVA IPG Bluestrip 3–10 NL/7cm). Rehydration was carried out at room temperature for 16 hrs. with protein sample diluted in rehydration buffer (8M Urea, 0.002% bromophenol blue, 2% w/v CHAPS, 3 mg dithiothretol, 1.5% ampholyte pH 3–10). For first dimensional electrophoresis, IPG strips were transferred to Hoefer IPG phor II. Iso electro focusing (IEF) was then performed at 20°C by a series of steps as follows: constant 250 V, 1:00 hrs.; constant 500 V, 1:00 hrs.; gradient 1000 V, 1:00 hrs.; gradient 3000 V, 2:00 hrs.; constant 3000 V, 2:00 hrs. The strips were then equilibrated in SDS equilibration buffer (6M Urea, 2% Tris HCl, 0.002% Bromophenol Blue) for 20 min. and loaded onto a 14% acrylamide gel for second dimension resolution. Second dimension run was carried out with mini gel SE 260 at 4V/cm. The 2DE protein spots were visualised by staining with 0.2% silver nitrate solution.

### Nano-liquid chromatography mass spectrometry (nLC-MS/MS)

The details of nLC-MS/MS analysis has been described elsewhere^44^ and used with a minor modifications for PTM analysis.

- Protein digestion

The silver stained protein spots in gels were excised carefully with help of a sterilized spatula and transferred into separate vials. Destaining and acetone precipitation was carried out. In-gel digestion was performed; samples were reduced with 5 mM TCEP (Tris 2-Carboxyethyl Phosphine) at 55°C for 1 hour and further alkylated with 50 mM iodoacetamide for 30 min at room temperature in the dark. The gel spots were shrinked with acetonitrile and air-dried for few minutes at room temperature followed by digestion with trypsin (1:50, trypsin/lysate ratio) for 16 hours at 37°C. Digests were dried using speed vac for 1 hour and pellet was dissolved in buffer A (5% acetonitrile, 0.1% formic acid). Digests were cleaned by Sep-Pak. Titanium method was performed for phospho binding. Digests were cleaned up again using C18 silica cartridge (The Nest Group, Southborough, MA) following manufacturer’s protocol and dried using speed vac. The desalted dried pellet was reconstituted in buffer A (5% acetonitrile, 0.1% formic acid).

b. Liquid chromatography mass spectrometry analysis

All the experiments were performed using EASY-nLC 1000 system (Thermo Fisher Scientific) coupled to Q Exactive mass spectrometer (Thermo Fisher Scientific, Germany) equipped with nano electrospray ion source. Peptide mixture (1.0 µg) was loaded on a precolumn and was resolved using 5 cm PicoFrit column (360 µm outer diameter, 75 µm inner diameter, 10 µm tip) filled with 1.9 µm of C18-resin (Dr Maeisch, Germany). The peptides were loaded with buffer A and eluted with a 0–40% gradient of buffer B (95% acetonitrile, 0.1% formic acid) at a flow rate of 500 nl/min for 10 min. The QExactive was operated using the Top10 HCD data-dependent acquisition mode with a full scan resolution of 70,000 at m/z 400. MS/MS scans were acquired at a resolution of 17500 at m/z 400. Lock mass option was enabled for polydimethylcyclosiloxane (PCM) ions (m/z = 445.120025) for internal recalibration during the run. MS/MS data was acquired using a data-dependent top 10 method dynamically choosing the most abundant precursor ions from the survey scan.

c. Protein identification and PTM analysis

The .raw files generated were analyzed using Proteome Discoverer (v2.2) against the in-house Uniprot reference proteome database (*Capra hircus, Ovis aries, Homo sapiens* and *Bos taurus*). Due to scarcity of available protein annotations for goat, the analysis was carried out with goat, human, sheep and cow database with 7056, 20117, 27666, and 23869 entries respectively. For Sequest search, the precursor and fragment mass tolerances were set at 10–15 ppm and 0.5 Da, respectively. The protease used to generate peptides, that is the enzyme specificity was set for trypsin/P (cleavage at the C terminus of “K/R”: unless followed by “P”) along with maximum missed cleavages value of 2. Carbamidomethyl on cysteine as fixed modification and oxidation of methionine and N-terminal acetylation and phosphorylation (S, T, Y, D) were considered as variable modifications for database search. Peptide spectrum match and protein false discovery rate (FDR) were set to 0.01.

### Functional analysis

All the identified milk proteins were assigned their gene symbol via the Uniprot knowledgebase (http://www.uniprot.org/). Protein classification of the identified proteome and PTM genes subset were performed based on their functional annotations using Gene Ontology (GO) for biological process, subcellular localization and molecular function using the Database for annotation, visualization and integrated discovery (DAVID) version 6.8. The enrichment was performed with *Homo sapiens* database in background for the identified gene names and measured by fisher exact test in DAVID system.

The analysis for the PTM subset was performed using Cytoscape v.3.8.1. Protein interaction networks, biological pathways and protein clusters with *Homo sapiens* as reference database were generated using cluego+cluepedia plugin. Analyses were carried out with a significance level of 0.05 using a hypergeometric test and the Benjamini & Hochburg false discovery rate correction.

Protein interaction networks were analysed using search tool for the retrieval of interacting genes/proteins (STRING version 11.0). STRING networks were calculated at highest confidence score of 0.900 for the entire set of milk proteins and for PTM protein subsets and interactions were clustered using MCL algorithm. The gene search for network was performed in *Homo sapiens* reference database.

The peptides showing modifications were grouped based on the length as small (<7AA), medium (7–25 AA) and long (>25AA). The functions of these peptides were determined by Milk bioactive peptide database (MBPDB)^69^ at threshold of 80% identity match for their known functions.

## Supplementary Information

Supplementary Information 1.

Supplementary Information 2.

Supplementary Information 3.

Supplementary Information 4.

Supplementary Information 5.
