# Supplementary material for: Post translational modifications of milk proteins in geographically diverse goat breeds
Source: Sci Rep. 2021 Mar 10;11:5619. doi: 10.1038/s41598-021-85094-9 (PMC7946870; doi:10.1038/s41598-021-85094-9)
Supplement: Supplementary file 1 — Supplementary Information 1. [file 41598_2021_85094_MOESM1_ESM.pdf]

Title: Post translational modifications of milk proteins in geographically diverse goat breeds

Authors: Pramod K. Rout[1]* and Mahima Verma[1]

[1]Genetics and Breeding Division, ICAR-Central Institute for Research on Goats, Makhdoom, Farah, Mathura, Uttar Pradesh. India - 281122.

***Corresponding author** –Dr. P. K. Rout

Principal Scientist

Genetics and Breeding Division,

ICAR-Central Institute for Research on Goats,

Makhdoom, Farah, Mathura, Uttar Pradesh. India - 281122.

E.mail: prashmirout@gmail.com, pramod.rout@icar.gov.in

Table S1: Description of goat breeds, their natural habitats and their utility

Breed/ Genotype	Specialty/ Utility	Agro-climatic zone	Home tract	Number of milk samples analysed by SDS-PAGE
**Jamunapari**	Milk	Semi-arid	Distributed mainly in Chakarnagar area of Etawah, Uttar Pradesh.	**668**
**Barbari**	Milk and Meat	Semi-arid	Distributed in western Uttar Pradesh, and adjoining districts of Rajasthan.	**242**
**Black Bengal**	Meat	Hot and Humid	Distributed in Eastern coastal region	**78**
**Gaddi**	Meat and Milk	Himalayan	Kangra and Kullu valley, Chamba, Sirmur, Simla and Lahaul and Spiti in Himachal Pradesh, hilly districts of Tehri Garhwal, Chamoli in Uttaranchal and in parts of Jammu hills.	**46**
**Sirohi**	Meat and Milk	Dry and Arid	Ajmer, Bhilwara, Tonk, and Jaipur in Rajasthan.	**90**
**Jakhrana**	Milk	Semi-arid	Distributed in villages near Behror Tehsil in Alwar, Rajasthan.	**45**
**Osmanabadi**	Meat and Milk	Semi-arid	Ahmednagar, Latur, Osmanabad, Solanpur districts of Maharashtra.	**20**
**Ganjam**	Meat	Hot, Humid and Coastal	Found in the Ganjam district of Odisha.	**10**

**Attappady Black**	Meat	Sub-humid and semi-arid	These goats are found exclusively in Attappady, an isolated hilly region of Kerala, India.	**13**
**Himalayan local**	Meat	Humid and cold	Native to the Himalayas in southern Tibet, northern India and Nepal	**28**
**Total**				**1240**

Table S6: GO terms, associated genes identified in network by cytoscape Cluego analysis of PTM subset

GOID	Ontology category	GO Term	Associated Genes Found	GO Levels	GO Groups	No. Genes	% Associated Genes	Term PValue	Term PValue Corrected with Bonferroni step down	Group PValue	Group PValue Corrected with Bonferroni step down	Over View Term
GO:0005200	GO_BiologicalProcess	**structural constituent of cytoskeleton**	[KRT14, KRT6A, KRT6B, KRT9, NEFH, VIM]	[6]	4	6	5.555555	3.20E-06	4.81E-05	Group4=2.326E-5	Group4=1.163E-4	FALSE
GO:0006334	GO_BiologicalProcess	**nucleosome assembly**	[H2BC12, H2BC21, H2BU1, H3C1, H3C13]	[6, 7, 8, 9]	3, 5	5	3.401361	2.29E-04	0.00206	Group3=1.381E-5, Group5=0.001	Group3=8.286E-5, Group5=0.003	FALSE
GO:0007595	GO_BiologicalProcess	**lactation**	[CAD, CSN2, CSN3]	[5, 6, 7, 8]	0	3	4.83871	0.00164	0.00656	Group0=0.001	Group0=0.005	TRUE
GO:0031424	GO_BiologicalProcess	**keratinization**	[KRT1, KRT10, KRT14, KRT27, KRT6A, KRT6B, KRT9]	[2, 6, 7, 8]	4	7	3.017242	2.61E-05	3.40E-04	Group4=2.326E-5	Group4=1.163E-4	FALSE
GO:0042542	GO_BiologicalProcess	**response to hydrogen peroxide**	[HBA1, LCN2, LDHA, ND5, ND6]	[4, 5]	1	5	3.184713	3.11E-04	0.002485	Group1=3.106E-4	Group1=0.001	TRUE
GO:0045104	GO_BiologicalProcess	**intermediate filament cytoskeleton organization**	[KRT14, KRT6A, KRT9, NEFH, VIM]	[3, 6]	4	5	9.259259	1.78E-06	3.02E-05	Group4=2.326E-5	Group4=1.163E-4	FALSE
GO:	GO_Biolo	**intermediate**	[KRT14,	[4, 5,	4	4	15.3846	2.57E-	4.11E-05	Group4	Group4=1	FALS

0045 109	gicalProce ss	**filament organization**	KRT9, NEFH, VIM]	7]			2	06		=2.326E -5	.163E-4	E
GO: 0045 778	GO_Biolo gicalProce ss	**positive regulation of ossification**	[BMPR2, NPPC, ZBTB16]	[3, 4, 5]	2	3	3.15789 5	0.00549 6	0.005496	Group2 =0.005	Group2=0 .005	TRUE
GO: 0050 830	GO_Biolo gicalProce ss	**defense response to Gram-positive bacterium**	[H2BC12, H2BC21, HAVCR2, KRT6A, LALBA]	[5, 6, 7]	3	5	4.62963	5.35E- 05	6.43E-04	Group3 =1.381E -5	Group3=8 .286E-5	TRUE
GO: 0061 844	GO_Biolo gicalProce ss	**antimicrobial humoral immune response mediated by antimicrobial peptide**	[H2BC12, H2BC21, KRT6A]	[4, 5, 6]	3, 5	3	3.79746 8	0.00327 7	0.006554	Group3 =1.381E -5, Group5 =0.002	Group3=8 .286E-5, Group5=0 .004	FALS E
GO: 0070 268	GO_Biolo gicalProce ss	**cornification**	[KRT1, KRT10, KRT14, KRT27, KRT6A, KRT6B, KRT9]	[3, 4, 7, 8, 9]	4	7	6.03448 3	2.62E- 07	4.72E-06	Group4 =2.3265 E-5	Group4=1 .163E-4	TRUE
KEG G:04 915	KEGG	**Estrogen signaling pathway**	[FKBP4, KRT10, KRT14, KRT27, KRT9]	[-1]	4	5	3.62318 9	1.71E- 04	0.001707	Group4 =2.326E -5	Group4=1 .163E-4}	FALS E
KEG G:05 034	KEGG	**Alcoholism**	[H2BC12, H2BC21, H2BU1, H3C1, H3C13, H3C15]	[-1]	5	6	3.20855 6	7.27E- 05	8.00E-04	Group5 =0.002	Group5=0 .003	FALS E
KEG G:05	KEGG	**Staphylococcus aureus infection**	[KRT10, KRT14,	[-1]	4	4	4.16666 7	4.68E- 04	0.002341	Group4 =2.326E	Group4=1 .163E-4	FALS E

150			KRT27, KRT9]							-5		
KEGG:05322	KEGG	**Systemic lupus erythematosus**	[H2BC12, H2BC21, H2BU1, H3C1, H3C13, H3C15]	[-1]	5	6	4.411765	1.21E-05	1.70E-04	Group5=0.002	Group5=0.004	TRUE
WP:2369	WikiPathways	**Histone Modifications**	[H3C1, H3C13, H3C15]	[-1]	5	3	4.285714	0.002324	0.006971	Group5=0.002	Group5=0.004	FALSE
WP:4320	WikiPathways	**The effect of progerin on the involved genes in Hutchinson-Gilford Progeria Syndrome**	[H3C1, H3C13, H3C15]	[-1]	5	3	7.692308	4.22E-04	0.002529	Group5=0.002	Group5=0.004	FALSE
WP:4553	WikiPathways	**FBXL10 enhancement of MAP/ERK signaling in diffuse large B-cell lymphoma**	[H3C1, H3C13, H3C15]	[-1]	5	3	7.894737	3.90E-04	0.002731	Group5=0.002	Group5=0.004	FALSE

Table S7: Pathways in signal transduction and associated proteins found in Reactome analysis of phosphoprotein subset

Pathway identifier	Pathway name	#Entities found	Entities ratio	Entities pValue	Entities FDR	Associated Gene ID
R-HSA-449147	**Signaling by Interleukins**	14	0.043549	1.57E-04	0.001411	HIST2H3A;PTPRZ1;CFL1;H3FA;LCN2;VIM;BDP1;HIST2H3D;CCR2;HAVCR2
R-HSA-8939211	**ESR-mediated signaling**	8	0.017447	4.69E-04	0.002831	CREBBP;HIST2H3A;HIST1H2BK;H3FA;HIST3H2BB;HIST2H3D;FKBP4;HIST2H2BE
R-HSA-9006931	**Signaling by Nuclear Receptors**	8	0.026239	0.005883	0.023533	CREBBP;HIST2H3A;HIST1H2BK;H3FA;HIST3H2BB;HIST2H3D;FKBP4;HIST2H2BE
R-HSA-201681	**TCF dependent signaling in response to WNT**	7	0.014721	8.66E-04	0.005052	CREBBP;HIST2H3A;HIST1H2BK;H3FA;HIST3H2BB;HIST2H3D;HIST2H2BE
R-HSA-157118	**Signaling by NOTCH**	7	0.017583	0.002369	0.011229	CREBBP;HIST2H3A;HIST1H2BK;H3FA;HIST3H2BB;HIST2H3D;HIST2H2BE
R-HSA-195721	**Signaling by WNT**	7	0.022627	0.009118	0.027496	CREBBP;HIST2H3A;HIST1H2BK;H3FA;HIST3H2BB;HIST2H3D;HIST2H2BE
R-HSA-194315	**Signaling by Rho GTPases**	7	0.031146	0.042419	0.11146	HIST2H3A;CFL1;HIST1H2BK;H3FA;HIST3H2BB;HIST2H3D;HIST2H2BE
R-HSA-1266695	**Interleukin-7 signaling**	2	0.002113	0.020362	0.061086	HIST2H3A;H3FA;HIST2H3D
R-HSA-9008059	**Interleukin-37 signaling**	2	0.002453	0.026848	0.080544	BDP1
R-HSA-1251985	**Nuclear signaling by ERBB4**	2	0.003203	0.043558	0.11146	CSN2
